# PRIME: a database for 16S rRNA microbiome data with phenotypic reference and comprehensive metadata

**DOI:** 10.1093/nar/gkaf1057

**Published:** 2025-10-31

**Authors:** Zhizhuo Zhang, Hongyu Zhao, Tao Wang

**Affiliations:** Department of Bioinformatics and Biostatistics, School of Life Sciences and Biotechnology, Shanghai Jiao Tong University, Shanghai 200240, China; SJTU–Yale Joint Center for Biostatistics and Data Science, National Center for Translational Medicine, Shanghai Jiao Tong University, Shanghai 200240, China; Department of Biostatistics, Yale University, New Haven, CT 06511, United States; Department of Bioinformatics and Biostatistics, School of Life Sciences and Biotechnology, Shanghai Jiao Tong University, Shanghai 200240, China; SJTU–Yale Joint Center for Biostatistics and Data Science, National Center for Translational Medicine, Shanghai Jiao Tong University, Shanghai 200240, China; Department of Statistics and MOE-LSC and CMA-Shanghai, School of Mathematical Sciences, Shanghai 200240, China; MoE Key Lab of Artificial Intelligence, AI Institute, Shanghai Jiao Tong University, Shanghai 200240, China

## Abstract

PRIME (Phenotypic Reference for Integrated Microbiome Enrichment) is a curated and standardized database of human microbiome 16S rRNA amplicon sequencing data, designed to facilitate cross-study analysis, reproducibility, and phenotype-driven discovery. PRIME aggregates 53 449 samples from 111 public studies, covering 93 body sites and 101 phenotypic categories, with detailed harmonization of sample-level metadata such as disease status, demographics, body sites, sequencing protocols, and experimental design. Each sample includes taxonomic abundance profiles generated via a consistent pipeline using both SILVA (138.2) and Greengenes2 (2024.09) reference databases, with results reported at multiple taxonomic levels as observed abundances (read counts) and relative abundances (proportions). A major strength of PRIME is its extensive manual curation, which standardizes phenotypic and contextual metadata across studies, enabling precise querying and robust phenotype-based comparisons. Users can interactively explore the database through a modern web interface, filter and visualize data by metadata fields, and download customized subsets. Programmatic access is supported via RESTful APIs and R package. PRIME aims to advance microbiome data integration and is continuously updated to incorporate new studies and features. The database is freely available at https://primedb.sjtu.edu.cn.

## Graphical Abstract

**Graphical Abstract:**
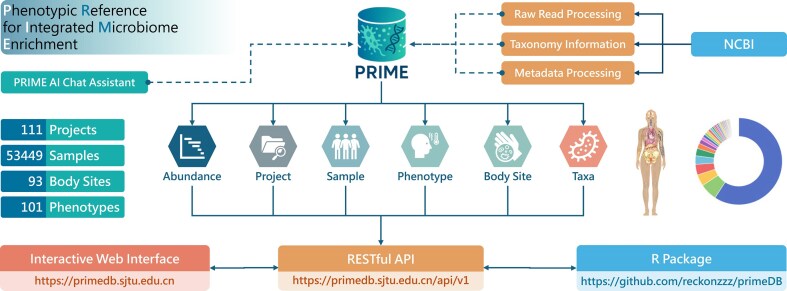

## Introduction

Over the past decade, microbiome research has become an increasingly prominent area in biomedical science, providing valuable insights into the potential roles of microbial communities in human health and disease [1–6]. Among the various high-throughput techniques used to study the microbiome, 16S rRNA amplicon sequencing remains one of the most widely adopted methods due to its cost-effectiveness, taxonomic resolution, and compatibility with large-scale population studies [7–9], such as the Human Microbiome Project [10, 11] and the Earth Microbiome Project [12]. Consequently, extensive collections of human-associated 16S rRNA sequences have been deposited in public repositories including the NCBI Sequence Read Archive (SRA) [13], the European Nucleotide Archive [14], and the DNA Data Bank of Japan [15].

Although public databases serve as a critical resource for microbiome research, their utility in detailed 16S rRNA analyses can be hindered by practical and technical limitations, including inconsistencies in experimental protocols, sequencing platforms, metadata annotation, and terminological conventions across studies [7, 16, 17]. Inconsistent metadata, especially related to phenotypes, body sites, protocols, and study designs, poses a major barrier to data integration, meta-analysis, and reproducible machine learning in microbiome research [18, 19]. While several databases aggregate microbiome data from individual studies [20–22], many lack standardized metadata, support for programmatic access, interactive visualization, or the ability to retrieve abundance tables. These gaps highlight the need for a high-quality, phenotypic microbiome resource that integrates 16S rRNA amplicon sequencing data across studies with harmonized metadata.

Here, we developed PRIME (**P**henotypic **R**eference for **I**ntegrated **M**icrobiome **E**nrichment), an openly accessible, manually curated database with programmatic access via RESTful APIs and R package. PRIME aggregates and standardizes human-associated 16S rRNA amplicon sequencing data, accompanied by detailed phenotypic, anatomical, and contextual metadata. It employs a uniform bioinformatic pipeline and harmonizes sample-level annotations to ensure consistency and comparability across studies. PRIME also adheres to the FAIR principles (findability, accessibility, interoperability, and reusability [23]), enabling researchers to efficiently identify relevant samples, conduct cross-study analyses, and advance reproducible, integrative microbiome research.

## Materials and methods

### Data collection

To establish a curated and high-quality collection of human-associated 16S rRNA microbiome studies, we systematically searched the NCBI BioProject database (https://www.ncbi.nlm.nih.gov/bioproject) using the keywords “human microbiome” and “human metagenome,” aiming to identify publicly available studies suitable for standardized processing and integration. We manually screened the resulting studies, starting with the most recent ones and assessed each for inclusion based on relevance and the availability of usable metadata.

Studies were included if they met all of the following criteria: (i) sequencing data corresponded to 16S rRNA amplicon sequencing, as confirmed by both metadata and bioinformatic validation; (ii) at least 75% of the amplicon data within a study were derived from human samples; and (iii) sample-level metadata provided sufficient information, either directly or through reliable interpretation, to assign phenotypes to individual runs (see Supplementary Note S1 for details). To maintain consistency, we excluded runs corresponding to shotgun metagenomics or lacking phenotype-related metadata.

Following a systematic review of 350 candidate studies, we identified 135 that fulfilled the established inclusion criteria. To ensure consistency, we further restricted our selection to studies sequenced on Illumina platforms, which represented the majority of publicly available studies. After applying this filter, a total of 115 studies were selected for bioinformatic processing using our standardized pipeline. Of these, four studies were excluded due to quality control failures. A total of 111 high-quality human 16S rRNA sequencing studies were ultimately included in the PRIME database, comprising 53 449 samples. These samples are defined as individual run accessions, since all curation and analyses were conducted at the run level.

### Bioinformatic pipeline

All raw 16S rRNA amplicon sequencing data were downloaded and converted from the NCBI SRA database using the SRA Toolkit (https://github.com/ncbi/sra-tools). Primer and adapter sequences were removed using Cutadapt [24] when necessary. Read quality was assessed using FastQC (http://www.bioinformatics.babraham.ac.uk/projects/fastqc), and summaries were generated with MultiQC [25]. Processed reads were then imported using QIIME 2 (2024.5) [26]. Denoising was performed using the DADA2 plugin [27], with parameters optimized for each study based on read quality profiles and the expected overlap of the targeted variable regions. This process included quality filtering, length trimming, chimera removal, and inference of exact amplicon sequence variants (ASVs). We did not perform clustering into operational taxonomic units, as ASVs are increasingly accepted as a more accurate and standardized alternative for 16S rRNA data analysis [28]. Taxonomic assignment was conducted using pretrained QIIME 2 classifiers against both the SILVA (138.2) [29] and Greengenes2 (2024.09) [30] reference databases. Final outputs included ASV abundance tables at multiple taxonomic ranks (phylum to species), provided both as observed abundances (read counts) and relative abundances (proportions). In this study, only the observed abundances were used. All software employed in the pipeline is listed in Supplementary Table S1.

### Metadata curation and standardization

Original sample-level metadata for all included studies were retrieved from the NCBI SRA Run Selector (https://www.ncbi.nlm.nih.gov/Traces/study). For each study, we manually confirmed the associated publications where available. Descriptive fields from the SRA metadata, study abstracts, and publications were systematically parsed to extract interpretable metadata elements. This was achieved through a combination of regular expression-based parsing and manual annotation, allowing free-text sample descriptions and custom-defined fields to be interpreted into structured metadata fields.

To ensure consistency across studies, equivalent metadata fields were standardized using a controlled vocabulary (see Supplementary Table S3). Manual harmonization was performed to unify terms referring to the same concept (e.g. synonymous disease labels, body site terms, or experimental conditions), followed by a secondary round of manual verification to ensure accuracy and uniformity. Furthermore, each sample was categorized by body sites into 1 of 10 high-level human systems: digestive system/gut, reproductive system, skin system, respiratory system, oral cavity system, mother–infant interface, cardiovascular system, nervous system, urinary system, and visual system (details in Table 1). Samples from unknown human body sites were labeled as unknown system, and those derived from environmental or non-human sources were assigned as non-human.

**Table 1.** Summary of body sites categorized by systems as defined in PRIME database

System	Representative body sites
Digestive system	Stool/feces, adjacent non-tumor liver tissue, hepatocellular carcinoma, stomach, intraductal papillary mucinous neoplasm, rectum, colon, ileum, squamous tissue, ..
Reproductive system	Vagina, penis, semen, perineum swab, ovary, cervical cancer tissue
Skin system	Skin, hand skin, gluteal crease skin, facial skin, middle toe skin, big toe skin, scalp, nose skin, axillary vault skin, external auditory canal skin, human axillary vault
Respiratory system	Oropharyngeal swab, nasopharynx, nasal cavity, nasal lavage fluid, sputum, lung swab, bronchoalveolar lavage fluid, ..
Oral cavity system	Oral cavity, oral wash, oral swab, dental plaque, saliva, tongue, ..
Mother–infant interface	Breast milk, colostrum, breast cancer tissue, adjacent non-tumor breast tissue (breast cancer), benign breast tissue (breast cancer)
Cardiovascular system	Blood, blood vessel, mitral valve, aortic valve, tricuspid valve
Nervous system	Brain tumor tissue, vagal paraganglioma tissue, carotid paraganglioma tissue, adjacent non-tumor brain tissue (brain tumor), head and neck cancer tissue
Urinary system	Urine, prostate cancer tissue, expressed prostatic secretion (EPS), prostate tissue, adjacent non-tumor prostate tissue (prostate cancer)
Visual system	Eye, conjunctiva

Body sites with ellipses (..) indicate partial listings due to space limitations; full lists are available in the database.

For downstream filtering and analytical flexibility, each study was manually classified by experimental design to indicate whether it contained time series, matched-pair, or comparison data. In addition, the targeted variable regions (e.g. V4, V3–V4, or V4–V5) [31] were accurately determined for 94.3% of the samples, either by extracting from the associated publications or by inferring from primer sequences using a custom script. To support data reproducibility in accordance with FAIR principles, we recorded key parameters used in Cutadapt and DADA2 for every sample. We also evaluated and annotated the data quality for each study using a five-level scoring scheme (excellent, good, acceptable, poor, very poor), based on raw read quality profiles and the proportion of input reads retained after DADA2 processing.

Ultimately, metadata fields curated in the PRIME database include, but are not limited to, sampling sites (systems, body sites), host metadata (phenotypes, BMI values, sex, ages, lifestyle factors), study designs (time series, comparison, matched), geographical details (continents, countries or regions), sequencing parameters (instruments, read layouts, sequencing lengths, targeted variable regions), bioinformatic parameters (software settings, data quality scores), and study-level information (associated publication DOIs, titles, and study descriptions).

### Taxonomic identifier mapping

As part of taxonomic standardization, we attempted to map every annotated taxon in PRIME to corresponding entries in the NCBI Taxonomy database [32]. We first employed the R package taxize [33] to match taxon names directly against NCBI Taxonomy records. However, due to naming inconsistencies, synonym usage, and formatting variations across reference databases, a substantial proportion of taxa could not be mapped through direct matching alone.

To address this, we developed a custom taxon-matching procedure that performed fuzzy and wildcard-based string matching against a locally cached, comprehensive NCBI Taxonomy table (see Supplementary Note S2 for details). This approach enabled partial or approximate name alignment for many previously unmatched taxa. All successfully matched taxa were subsequently subjected to manual verification via the official NCBI Taxonomy website to ensure correctness. This combination of automated and manual curation ensured accurate and unambiguous linkage of PRIME taxonomic annotations to standardized NCBI Taxonomy identifiers.

### Architecture and implementation of PRIME database

The PRIME database is implemented as a modern, user-friendly and full-stack web application following a decoupled frontend–backend architecture. The frontend is built using Vue 3 and Vite, delivering a high-performance, modular, and responsive user interface that seamlessly adapts to desktops, tablets, and mobile devices. Cross-device compatibility has been validated through extensive testing (details in Supplementary Table S2). Interactive charts and dynamic filtering components are integrated throughout the site to support intuitive and flexible data exploration. To help users better understand the database, we additionally deployed an AI-powered chat assistant using the Dify platform (https://github.com/langgenius/dify). This assistant operates through a knowledge-based workflow and is capable of answering questions related to PRIME’s features, structure, and usage.

The backend is developed in Node.js using the Express.js web framework, with MySQL 8.0 serving as the primary relational database for structured storage of samples, metadata, taxonomic profiles, and related information. In-memory caching is handled via node-cache to accelerate responses for frequently accessed API endpoints. All communication between the frontend and backend is secured using the HTTPS protocol, ensuring data integrity and confidentiality during user interaction.

PRIME provides programmatic access through a versioned RESTful API (v1) and an accompanying R package, *primeDB*, enabling users to query and retrieve data within external analysis workflows. The API reference, accessible via the “Help” page, includes an interactive “View Response” button that allows users to preview real-time responses. The *primeDB* package wraps a set of predefined functions that directly interact with the API.

## Results

### Overview of PRIME

PRIME is a curated database comprising 53 449 human-associated 16S rRNA sequencing samples from 111 publicly available studies. All data in PRIME are openly accessible without login or restriction. Unlike existing resources, PRIME places a central emphasis on comprehensive, accurate, and standardized sample-level metadata—enabling robust cross-study integration and phenotype-driven microbiome research. The database integrates three core data types: (i) manually curated sample-level metadata, (ii) taxonomic abundance tables generated through a unified bioinformatic pipeline, and (iii) external taxonomic annotations accurately mapped to NCBI Taxonomy identifiers. All underlying sequence and metadata records were sourced from NCBI and underwent extensive manual validation to ensure completeness, accuracy, and consistency across studies.

**Figure 1.**
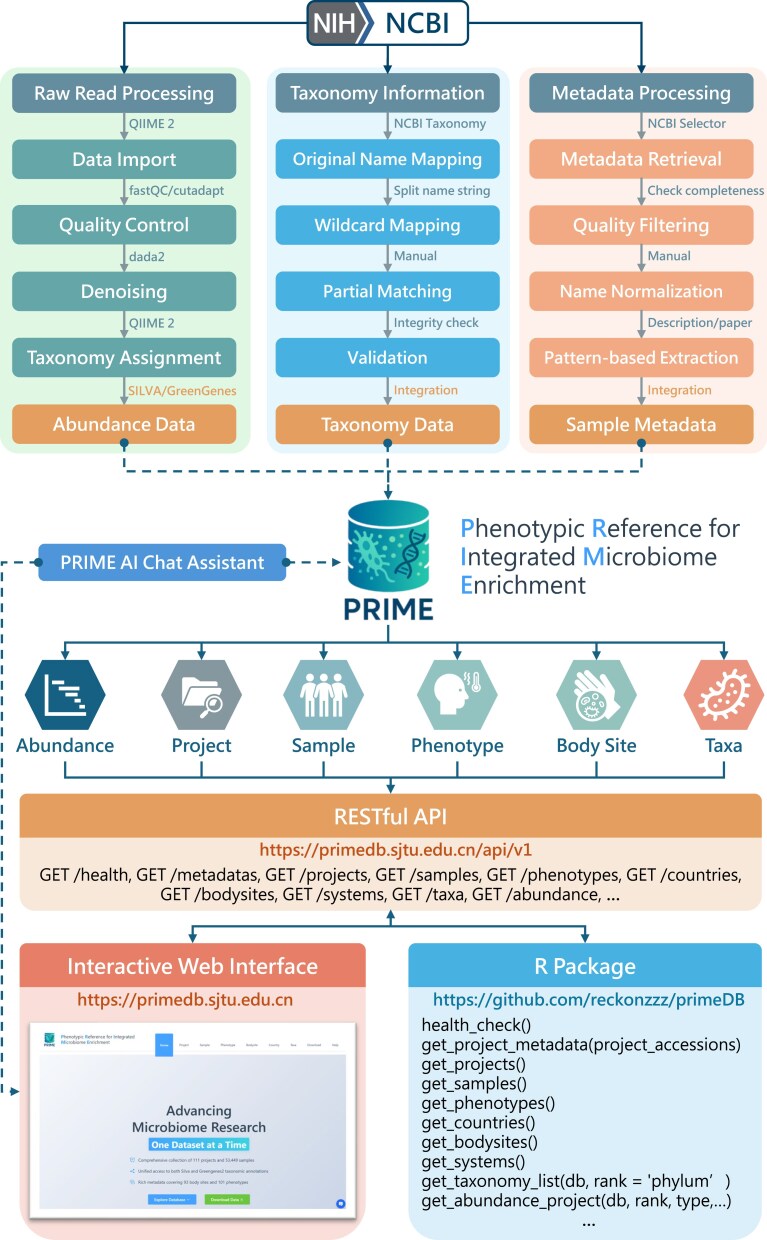
Schematic overview of the PRIME database architecture, data processing, and access modes. The diagram illustrates the processing of three core data types (top), six major database components (middle), and the three user access modes (bottom).

To support broad usability and flexible access, PRIME provides three complementary modes of interaction: a modern, user-friendly web interface; a standardized RESTful API; and an R package for seamless integration into custom workflows. Both the web interface and R package retrieve data via the backend API, ensuring consistent, secure, and efficient data delivery (Fig. 1). Although most data types are accessible through all access modes, the web interface offers the most intuitive and comprehensive platform for data exploration and retrieval.

The web platform offers six major functionalities: (i) full browsing, searching, filtering, and download of project- and sample-level metadata; (ii) phenotype-level summaries and sample selection; (iii) anatomical overviews based on system-level body site groupings; (iv) geographic exploration by continent, country or region; (v) taxonomy-based navigation via a hierarchical phylogenetic tree; and (vi) flexible download options for taxonomic abundance tables at multiple taxonomic levels, available as observed abundances (read counts) or relative abundances (proportions), with support for exporting either the full dataset or customized subsets.

### Interactive access via web interface

**Figure 2.**
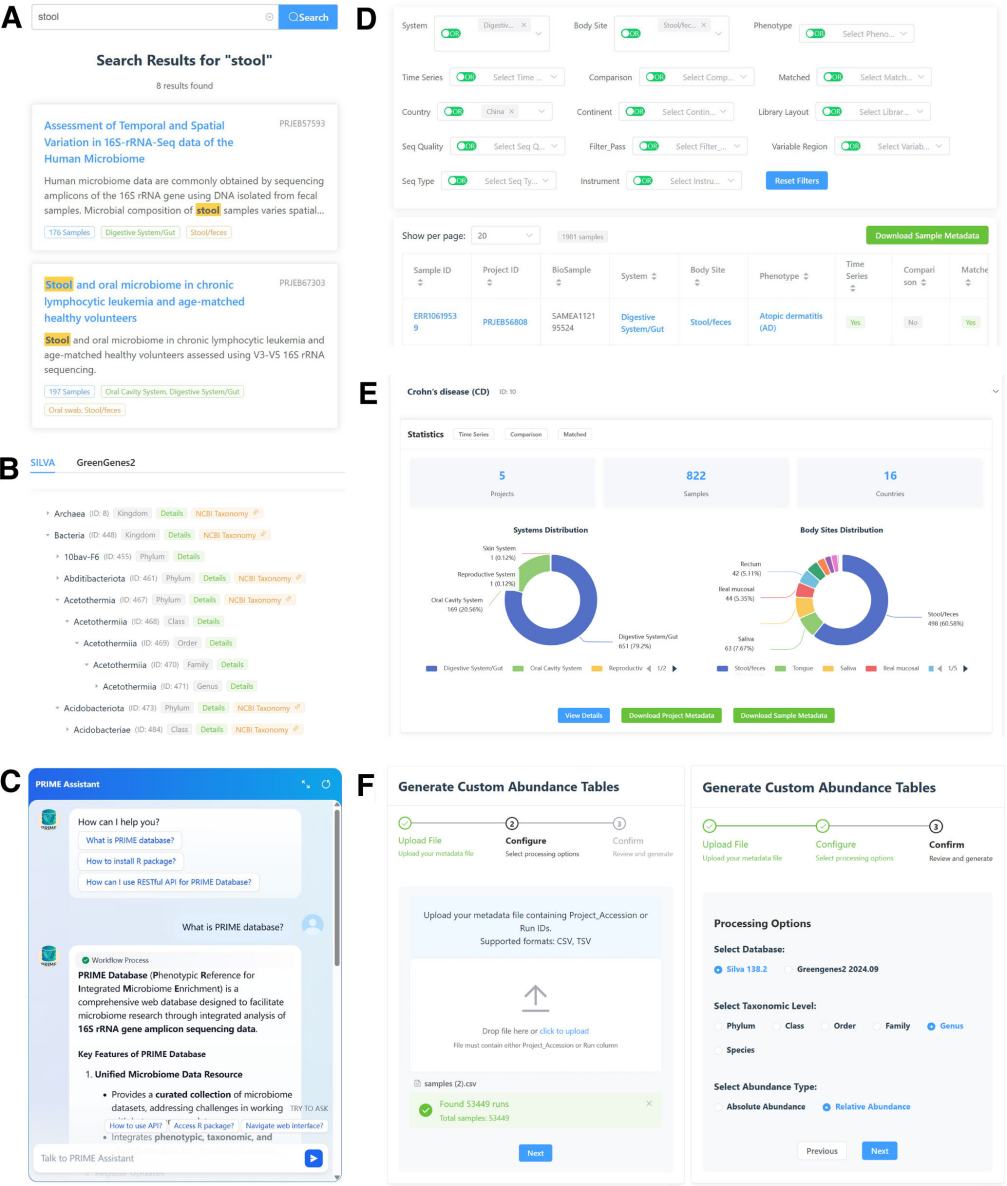
Representative features of the PRIME web interface. (**A**) Example of “Project” search results; the “Sample” page supports similar query functionality. (**B**) Taxonomic tree view on the “Taxa” page; clicking “Details” shows distribution patterns, and “NCBI Taxonomy” links to the corresponding page. (**C**) Demonstration of the PRIME AI assistant, powered by a predefined knowledge-based workflow. (**D**) Sample filtering interface on the “Sample” page; the “Project” page provides similar filtering capabilities. (**E**) Phenotype-specific detailed view accessed from the “Phenotype” page. (**F**) Custom abundance table generation interface, allowing users to upload project/sample lists, configure parameters, and submit asynchronous requests for tailored data.

The web interface provides an interactive platform for accessing and exploring the PRIME database. The homepage presents summary statistics and offers direct navigation to other pages. A floating button in the lower-right corner activates a built-in AI assistant for real-time guidance. The interface comprises seven main functional pages—“Project,” “Sample,” “Phenotype,” “Bodysite,” “Country,” “Taxa,” and “Download”—through which users can search, preview, filter, and download metadata and taxonomic abundance tables (examples in Fig. 2A–E). The “Help” page provides comprehensive documentation of the database, and each page also includes an expandable help panel. In summary, the web interface provides comprehensive access to the database, fulfilling the majority of users’ data retrieval and exploration needs.

### Programmatic access via RESTful APIs and R package

PRIME supports programmatic access via a versioned RESTful API (v1) and an accompanying R package, *primeDB*. The API adheres to standard REST principles [34] and delivers data in a structured JSON format, allowing users to query projects, samples, metadata, taxa, phenotypes, countries, and abundance tables. The *primeDB* package wraps the API functionality into user-friendly R functions, enabling seamless access to PRIME data within the R environment without the need for direct HTTP handling. Comprehensive API documentation is available on the “Help” page. No authentication or rate limiting is currently enforced, ensuring open and unrestricted programmatic access. Together, these tools facilitate reproducible research and streamline integration into downstream bioinformatic workflows.

### Support for full and customized abundance table generation

PRIME supports both full-dataset and customized retrieval of taxonomic abundance tables and sample metadata across all access modes—the web interface, RESTful API, and *primeDB* R package. Users can download full metadata and abundance tables at multiple taxonomic ranks (phylum to species) for both SILVA and Greengenes2 reference databases. On the web interface, the “Download” page provides direct access to these datasets through regularly updated (every 24 h) cached files. Equivalent endpoints and functions are available via the API and R package.

**Figure 3.**
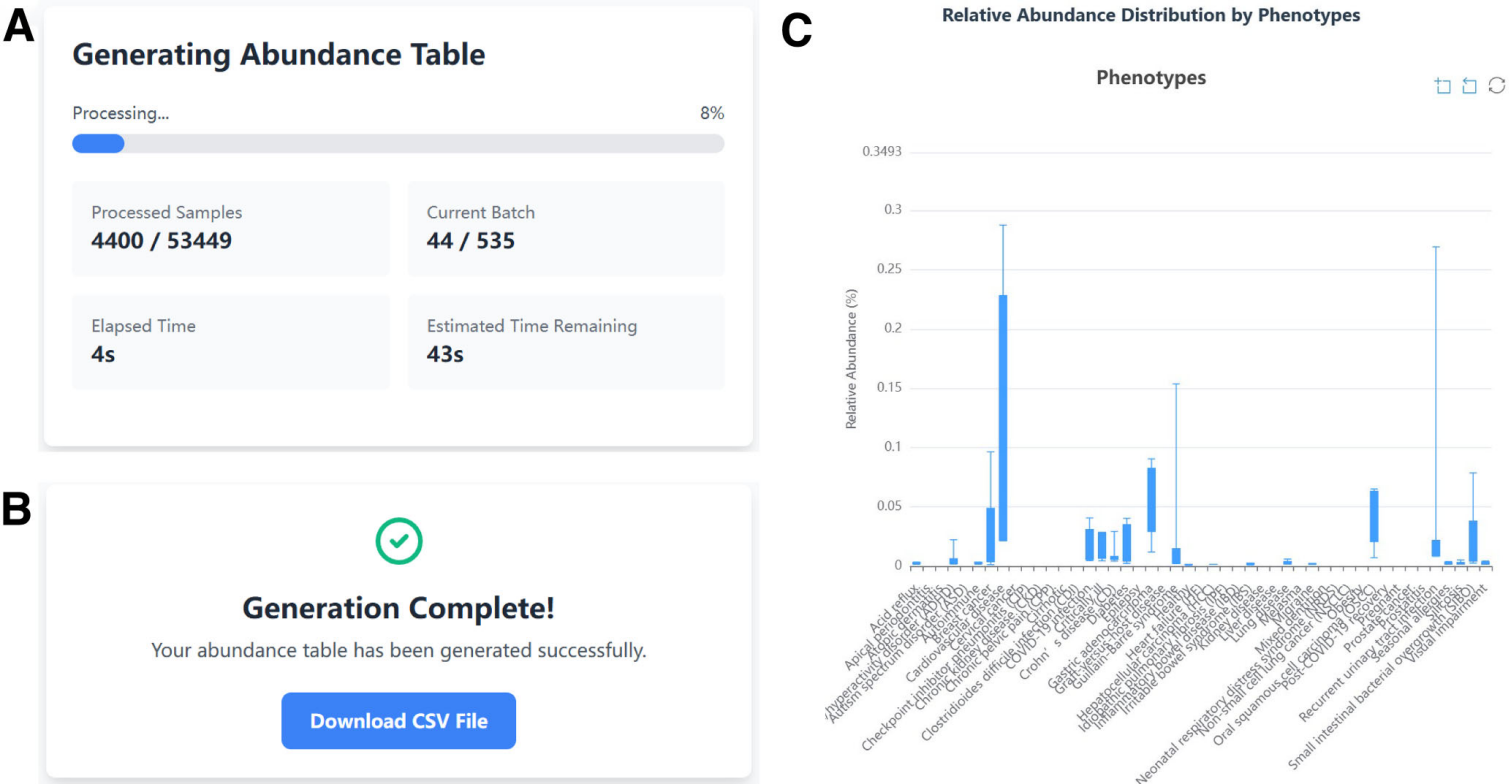
(**A**) Progress display for abundance table generation via a temporary download link. (**B**) Download interface for a completed custom abundance table file. (**C**) Example of taxon detail view: boxplots showing the relative abundance of *Elusimicrobiota* across phenotypes where it is present.

In addition to full downloads, PRIME enables users to generate customized abundance tables by specifying a subset of projects or samples. This functionality is available through the “Custom Abundance Generation” panel on the “Download” page, where users can upload a formatted file containing selected identifiers and configure output parameters (Fig. 2F). The backend automatically parses the input and initiates asynchronous generation of the requested list. To accommodate longer processing times, a temporary link is issued for each request, allowing users to monitor progress and download the resulting files within 24 h (Fig. 3A and B). The API and *primeDB* package provide analogous functionality, delivering results via a streaming response for improved efficiency with large data subsets.

### Detail pages for enriched contextual information

The PRIME web interface includes dedicated detail pages for multiple data categories, including projects, samples, phenotypes, body sites, systems, taxa, and countries. Each page presents relevant metadata and interactive visualizations to support detailed and contextual exploration.

Project and sample detail pages display complete metadata alongside related samples or projects. For phenotypes, body sites, systems, and countries, the detail pages summarize all associated studies and samples and provide interactive pie charts showing sample distribution. For each taxon, the detail page reports the related samples and phenotypes, along with visualizations of its relative abundance across phenotypes and body sites (Fig. 3C).

Cross-links between detail pages—for example, from a sample to its related project or phenotype—are consistently implemented throughout the interface, enabling seamless navigation and integrated data exploration.

## Discussion

### Comparison with existing resources

A number of public databases and resources have been developed to support microbiome research, each addressing distinct aspects of microbiome data integration, annotation, and accessibility. These resources vary in terms of data type (e.g. 16S rRNA amplicon sequencing versus shotgun metagenomics), anatomical or phenotypic coverage, richness of metadata curation, and modes of data access. Representative databases include the following.

**GMrepo** provides a manually curated database focusing primarily on the human gut microbiome and includes both 16S and shotgun metagenomic data organized by phenotype [22]. Similarly, **mBodyMap** catalogs microbial distributions across various human body sites, with an emphasis on anatomical diversity [21]. Both databases represent valuable resources for the microbiome community; however, they currently do not provide functionality to export sample-level abundance tables. **MGnify**, maintained by EMBL-EBI, is a large-scale analysis platform supporting multiple sequencing types through automated pipelines, providing processed results for a wide range of environmental and host-associated microbiomes [35]. This platform offers extensive coverage and automated processing, but its reliance on submitter-provided metadata—often unstandardized or incomplete—can limit its utility for cross-study comparisons. The **Human Microbiome Compendium** (**HMC**) demonstrates the utility of uniform processing at scale, having applied a consistent pipeline to over 168 000 16S rRNA sequencing samples in human gut [20]. While HMC highlights the potential of uniform large-scale processing, the lack of per-sample error control, curated metadata, and interactive features can limit its usefulness for in-depth comparative studies. In contrast, **curatedMetagenomicData** offers structured and harmonized human microbiome datasets within the R/Bioconductor ecosystem [36]. While it provides well-annotated metadata and programmatic access, it focuses exclusively on shotgun metagenomics.

We extend these efforts by focusing specifically on human-associated 16S rRNA sequencing studies and addressing a key unmet need: the integration of large-scale, sample-level metadata that is manually curated, standardized across studies, and directly linked to consistently processed taxonomic abundance tables. Unlike most existing databases, PRIME supports both comprehensive and customizable data retrieval via an interactive web portal, a RESTful API, and an R package. Together, these features position PRIME as a complementary and user-centered resource within the broader microbiome data ecosystem.

### Future plans

PRIME is designed as a long-term, actively maintained resource. It is hosted on the jCloud platform at Shanghai Jiao Tong University, which provides institutional infrastructure and sustained support. Regular updates will incorporate newly released human 16S rRNA sequencing studies to expand coverage across body sites, phenotypes, and contextual metadata. To ensure sustainability, curation workflows have been documented, the bioinformatics pipeline has been automated, and team members are being trained to support ongoing maintenance. All updates will be versioned and documented to preserve data provenance and reproducibility.

## Conclusion

In this study, we presented PRIME, a curated and standardized database of human-associated 16S rRNA amplicon sequencing data, designed to enable phenotype-driven, cross-study microbiome research. PRIME currently contains 53 449 samples across 111 studies, covering 93 body sites and 101 phenotypic categories, with harmonized metadata and consistently processed taxonomic abundance tables using both SILVA and Greengenes2 reference databases. A major strength of PRIME lies in its extensive manual curation of metadata, facilitating integrative analyses across heterogeneous studies. In addition to its interactive, modern web interface, PRIME offers flexible programmatic access via a RESTful API and an R package, promoting reproducibility and seamless integration into external workflows.

PRIME fills a critical gap in the microbiome data ecosystem by providing easy access to high-quality, metadata-rich 16S rRNA datasets at scale. Moving forward, we will continue to expand the database with newly released studies. We believe that PRIME will serve as a valuable and sustainable resource for researchers in microbiome science, and we are committed to maintaining and updating the database regularly.

## Supplementary Material

gkaf1057_Supplemental_File

## Data Availability

All sequence data and associated raw metadata used in the PRIME database are publicly available. The PRIME interactive web interface can be accessed at https://primedb.sjtu.edu.cn. The API (v1) is available at https://primedb.sjtu.edu.cn/api/v1, and the corresponding R package can be found at https://github.com/reckonzzz/primeDB. All files are freely available for academic use under the MIT license and permanently archived at https://doi.org/10.5281/zenodo.15711237.
