# Supplementary material for: PRIME: a database for 16S rRNA microbiome data with phenotypic reference and comprehensive metadata
Source: Nucleic Acids Res. 2025 Oct 31;54(D1):D255–63. doi: 10.1093/nar/gkaf1057 (PMC12807763; doi:10.1093/nar/gkaf1057)
Supplement: gkaf1057_Supplemental_File [file gkaf1057_supplemental_file.pdf]

PRIME: a database for 16S rRNA microbiome data with phenotypic reference and comprehensive metadata

SUPPLEMENTARY DATA

Note S1. Inclusion criteria details.
Note S2. Procedures for mapping annotated taxa to NCBI Taxonomy.
Table S1. Software and versions used in the PRIME pipeline.
Table S2. Cross-platform compatibility testing of the PRIME web interface.
Table S3. Controlled vocabulary for standardized metadata fields.

Note S1. Inclusion criteria details.

(i) Sequencing type determination

Sequencing type was identified primarily from the Assay Type metadata field, with only runs annotated as amplicon sequencing retained. Because several projects contain multiple sequencing modalities (e.g., shotgun metagenomics, 18S rRNA amplicons), sequencing type was further verified during bioinformatic processing to ensure inclusion of only 16S rRNA amplicon data.

(ii) Sample-level metadata requirement

Inclusion further required metadata that enabled reliable assignment of phenotypes at the sample level. Phenotypic categories were either explicitly annotated or could be deduced from a combination of metadata fields, project descriptions, and, when applicable, associated publications.

Illustrative examples:

- *PRJNA1216621*

In this study, sample identifiers followed a structured convention, such as F-Pxxx and F-Hxxx. The accompanying project description stated that the study compared fecal microbiota between 60 patients with heart failure (HF) and 30 healthy controls. The prefix F-P corresponded to HF patient samples, whereas F-H denoted healthy control samples; the numeric suffix (xxx) represented individual subject IDs. This consistent labeling, in conjunction with the project-level description, enabled unambiguous classification of each sample into the appropriate phenotype category.

- *PRJNA978360*

In this study, phenotypic and temporal information could be reconstructed by integrating multiple sources of annotation. The metadata field Ecotype specified the sampling site (e.g., oral, gut, nasal). Sample names, such as RMB86V1S, RMB57V5A, and RMB89V4F, contained embedded codes: the subject identifier (numeric component), the visit number (V1, V4, V7 corresponding to admission, discharge, and follow-up, respectively), and a site-specific suffix (S, F, A indicating sampling locations consistent with the Ecotype annotation). Additional details provided in the associated publication (DOI: 10.3389/fcimb.2023.1193113) confirmed this interpretation. Together, these elements allowed accurate assignment of samples to both clinical phenotype (respiratory syncytial virus [RSV] infection) and temporal context (admission, discharge, or follow-up).

Note S2. Procedures for mapping annotated taxa to NCBI Taxonomy.

To harmonize taxonomic annotations across reference databases, all taxa in PRIME were attempted to be mapped to the NCBI Taxonomy database. Direct matching was initially attempted using the R package *taxize*. However, inconsistencies in nomenclature, synonym usage, and formatting between reference databases and NCBI Taxonomy frequently impeded exact matching. Representative examples include:

- **Nomenclature differences**: *Aerophobota* in SILVA 138.2 corresponds to *Candidatus Aerophobota* in NCBI Taxonomy; likewise, *Sericytochromatia* in SILVA corresponds to *Candidatus Sericytochromatia* in NCBI Taxonomy.
- **Alias usage**: *PB19* in SILVA is an alias of *Phenoliferia psychrophila* in NCBI Taxonomy and therefore cannot be directly mapped.
- **Formatting variation**: *Candidatus_Methylomirabilis* in SILVA requires removal of the underscore to match *Candidatus Methylomirabilis* in NCBI Taxonomy.

To address these challenges, a custom multi-step mapping procedure was implemented:

1. **Automated prefix handling**: unmatched taxa were re-tested after automatically adding the prefix "Candidatus".
2. **Synonym-aware remapping**: for remaining unmatched taxa, names from SILVA and Greengenes2 (2024.09) were cross-referenced against a pre-downloaded table of NCBI Taxonomy IDs, names, and synonyms. Tokens were standardized by splitting on delimiters (e.g., space, underscore) and matched using wildcard operators. Longest Common Subsequence (LCS) similarity was then applied to retain the top three candidate matches.
3. **Manual validation**: all candidate matches were verified using the NCBI Taxonomy Browser, and only confirmed matches were retained.
4. **Unresolved taxa**: entries that could not be reliably assigned due to conflicting or incomplete nomenclature were left unmapped.

This approach ensured systematic and reproducible harmonization of taxa while maintaining high confidence in the mappings used for downstream analyses.

Table S1. Software and versions used in the PRIME pipeline.

Software	Version	Source
FastQC	0.12.1	https://www.bioinformatics.babraham.ac.uk/projects/fastqc/
MultiQC	1.29	https://seqera.io/multiqc/
Cutadapt	4.9	https://cutadapt.readthedocs.io/en/v4.9/
QIIME2	2024.5	https://library.qiime2.org/quickstart/amplicon
DADA2	plugin	https://benjjneb.github.io/dada2/<br>(dada2-plugin of QIIME2 was used in our paper)

Table S2. Cross-platform compatibility testing of the PRIME web interface.

Platform	Browser	Responsive UI	Chart Rendering	AI Assistant
**iOS (iPhone)**	**Safari**	√	√	√*
	**Chrome**	√	√	√*
**iOS (iPad)**	**Safari**	√	√	√
	**Chrome**	√	√	√
**Android (Mobile)**	**Edge**	√	√	√*
	**Firefox**	√	√	√*
	**Chrome**	√	√	√*
**Android (Tablet)**	**Edge**	√	√	√
	**Firefox**	√	√	√
	**Chrome**	√	√	√
**Windows**	**Edge**	√	√	√
	**Firefox**	√	√	√
	**Chrome**	√	√	√
**Mac**	**Safari**	√	√	√
	**Edge**	√	√	√
	**Firefox**	√	√	√
	**Chrome**	√	√	√

* AI Assistant sometimes displays too large due to the small page height when in landscape mode on mobile devices.

Table S3. Controlled vocabulary for standardized metadata fields.

Field	Description
Run	Unique sequencing run accession identifier
AvgSpotLen	Average spot length (bp) per sequencing read
Bases	Total bases generated in the run
BioProject	NCBI BioProject accession ID
BioSample	NCBI BioSample accession ID
Collection_Date	Date when the biological sample was collected
Experiment	Experiment accession
Country	Country of sample collection
Continent	Continent of sample collection
Instrument	Sequencing instrument/platform used
Library_Name	Name of sequencing library
Release_Date	Date when sequencing data was released
Create_Date	Date when sequencing data was created
Sample_Name	Name/identifier of the biological sample
SRA_Study	SRA study accession
Project_name	Self-defined study/project name
Time_series	Indicates whether study has time-series sampling
Comparison	Indicates whether study has comparison groups
Matched	Indicates whether study has matched samples
Systems	Self-defined system of sample collection
Body_Site	Anatomical body site of sample collection
Phenotype	Phenotype of the host subject
Doi	Digital Object Identifier of the associated publication
Library_Layout	Sequencing library layout
Primer_Cut	Whether perform primer trimming
Primer_Par	Primer trimming parameters
Denoise_Par	Denoising parameters for dada2
Collapse	Indicates whether collapsed into Silva or greengenes2
Sequencing_Quality	Overall sequencing quality
Filter_Pass	Overall run passing quality filtering
Variable_Region	16S rRNA variable region targeted
Sequencing_Type	Read length
Note	Additional notes or comments
Time_Point	Sampling time point
Study_Group	Group assignment within the study
Participant_Id	Unique identifier for participant
Host_Age	Age of host individual
Host_Sex	Sex of host individual
Host_Height	Height of host individual
Host_Weight	Weight of host individual
Host_BMI	Body Mass Index of host individual
Host_Menopausal_Status	Menopausal status of host (for female subjects)

Race_or_Ethnicity	Race or ethnicity of host individual
Host_Birth_Year	Year of birth of host individual
Host_Country_Birth	Country of birth of host individual
Smoke_Status	Smoking status of host individual
Drinking_Status	Alcohol drinking status of host individual
Diet_Type	Dietary habit type
Probiotic_Frequency	Frequency of probiotic intake
Teethbrushing_Frequency	Frequency of tooth brushing
Sleep_Duration	Average sleep duration
Tumor_Stage	Tumor stage (oncology)
Node_Stage	Lymph node stage (oncology)
Metastasis	Metastasis status
Antibiotic_Route	Route of antibiotic administration
Antibiotics_Use	Antibiotics usage status
Monobutyl_Phthalate_Presence	Presence of monobutyl phthalate biomarker
Monoethyl_Phthalate_Presence	Presence of monoethyl phthalate biomarker
Vaginal_pH	Measured vaginal pH
Antibiotic_Status	Status of antibiotic exposure
Anti_Fungal	Antifungal treatment status
ALL_Type	Acute lymphoblastic leukemia (ALL) type
Endo_C_Value	Endometriosis clinical value (C)
Endo_M_Value	Endometriosis marker value (M)
On_PPI	Proton pump inhibitor usage
Neoadjuvant	Neoadjuvant therapy status
Adjuvant	Adjuvant therapy status
Pepsin_D	Pepsin diagnostic value
Pepsin_Level	Pepsin level measurement
RSI_D	Reflux Symptom Index (diagnostic)
RSI_Score	Reflux Symptom Index score
Ihmc_medication_code	Medication code from IHMC
Post_Pre_Surgery	Post- or pre-surgery status
Abnormal_Uterine_Bleeding	Presence of abnormal uterine bleeding
Confirmed_Adenomyosis	Diagnosis of adenomyosis
Confirmed_Cysts	Diagnosis of cysts
Confirmed_Fibroids	Diagnosis of fibroids
Endo_Location	Location of endometriosis
Endo_Stage	Stage of endometriosis
Heavy_Menstrual_Bleeding	Presence of heavy menstrual bleeding
Irregular_Menstrual_Bleeding	Presence of irregular menstrual bleeding
Sperm_Concentration	Sperm concentration measurement
Sperm_Motility	Sperm motility measurement
Fertility	Fertility status
Dominant_Hand	Dominant hand of host individual
